# Diagnosis and management of non-clonal erythrocytosis remains challenging: a single centre clinical experience

**DOI:** 10.1007/s00277-021-04546-4

**Published:** 2021-05-19

**Authors:** Saša Anžej Doma, Eva Drnovšek, Aleša Kristan, Martina Fink, Matjaž Sever, Helena Podgornik, Tanja Belčič Mikič, Nataša Debeljak, Irena Preložnik Zupan

**Affiliations:** 1 Department of Haematology, University Medical Centre Ljubljana, Zaloška 7, 1000 Ljubljana, Slovenia; 2 Faculty of Medicine, University of Ljubljana, Korytkova 2, 1000 Ljubljana, Slovenia; 3 Medical Centre for Molecular Biology, Institute of Biochemistry and Molecular Genetics, Faculty of Medicine, University of Ljubljana, Vrazov trg 2, 1000 Ljubljana, Slovenia; 4 Faculty of Pharmacy, University of Ljubljana, Aškerčeva 7, 1000 Ljubljana, Slovenia

**Keywords:** Secondary erythrocytosis, Idiopathic erythrocytosis, Congenital erythrocytosis, Algorithm, Next-generation sequencing

## Abstract

Erythrocytosis has a diverse background. While polycythaemia vera has well defined criteria, the diagnostic approach and management of other types of erythrocytosis are more challenging. The aim of study was to retrospectively analyse the aetiology and management of non-clonal erythrocytosis patients referred to a haematology outpatient clinic in an 8-year period using a 3-step algorithm. The first step was inclusion of patients with Hb > 185 g/L and/or Hct > 0.52 in men and Hb > 165 g/L and/or Hct > 0.48 in women on two visits ≥ two months apart, thus confirming true erythrocytosis. Secondly, polycythaemia vera was excluded and secondary causes of erythrocytosis (SE) identified. Thirdly, idiopathic erythrocytosis patients (IE) were referred to next-generation sequencing for possible genetic background evaluation. Of the 116 patients, 75 (65%) are men and 41 (35%) women, with non-clonal erythrocytosis 34/116 (29%) had SE, 15/116 (13%) IE and 67/116 (58%) stayed incompletely characterized (ICE). Patients with SE were significantly older and had significantly higher Hb and Hct compared to patients with IE. Most frequently, SE was attributed to obstructive sleep apnoea and smoking. Phlebotomies were performed in 56, 53 and 40% of patients in the SE, IE, and ICE group, respectively. Approx. 70% of patients in each group received aspirin. Thrombotic events were registered in 12, 20 and 15% of SE, IE and ICE patients, respectively. Congenital erythrocytosis type 4 (ECYT4) was diagnosed in one patient. The study demonstrates real-life management of non-clonal erythrocytosis which could be optimized using a 3-step diagnostic algorithm.

## Introduction

Erythrocytosis is a condition of a true increase in red cell mass (RCM), manifested by high haemoglobin (Hb) concentration (> 165 g/L in men and > 160 g/L in women) and/or haematocrit (Hct) (> 0.49 in men and > 0.48 in women) according to the 2016 WHO criteria for the diagnosis of polycythaemia vera [1, 2]. Most authors agree that persistently elevated Hb > 185 g/L and Hct > 0.52 in men and Hb > 165 g/L and Hct > 0.48 in women with non-clonal background warrants further investigations [2–5]. Both parameters are affected by plasma volume and its reduction causes relative or false erythrocytosis. Absolute or true erythrocytosis is defined by RCM greater than 125% of predicted for age and sex [6]. Nevertheless, the knowledge of personal baseline values is of great importance for the interpretation of results [4, 5].

The causal diversity of erythrocytosis is wide and therefore requires complex diagnostic algorithms. It can have a primary or secondary cause, and both can have either congenital or acquired origin [2–4]. Primary erythrocytosis results from a defect in the erythroid bone marrow compartment, while secondary erythrocytosis results from a pathological condition outside the bone marrow, most often hypoxia or dysregulation in the oxygen-sensing pathway. The most common form of erythrocytosis is acquired secondary erythrocytosis (SE), caused by pulmonary, cardiac, or vascular disorders that lead to hypoxia or from an external cause of hypoxia [4]. The only primary cause of clonal erythrocytosis is polycythaemia vera (PV), almost always associated with a *JAK2* mutation (*JAK2* V617F or exon 12) [2, 4]. Genetic background is very rare in non-clonal erythrocytosis. Several mutations in nine different genes lead to eight types of congenital (familial) erythrocytosis (ECYT1-8). Mutations in the erythropoietin receptor (*EPOR*) are indicative of congenital erythrocytosis type 1 (ECYT1). Mutations in genes involved in oxygen sensing (*VHL, EGLN1, EPAS1, EPO*) cause ECYT 2, 3, 4, and 5, respectively [7]. Mutations in genes that affect Hb oxygen affinity (*HBB, HBA1,* and *HBA2, BPGM*) cause ECYT 6, 7 and 8, respectively [7, 8]. Furthermore, in the majority of congenital erythrocytosis, the molecular defect is not identified [9] and many patients lack a specific diagnosis [8]. If no cause is found, the patient is diagnosed with idiopathic erythrocytosis (IE) [10].

Patient referral to the haematologist is often based on repeated complete blood count (CBC) without a thorough history, physical examination, or additional laboratory testing. It is important to be familiar with the contemporary evaluation of erythrocytosis, utilizing algorithms for efficient testing in the majority of patients in everyday clinical practice [2–5, 11]. Whereas PV is almost always easily confirmed, other types of erythrocytosis are often less well recognized, weakly proven or even ignored [12–16].

The aim of our study was to retrospectively analyse non-clonal erythrocytosis patients from our centre medical records in an eight-year period. Furthermore, our goal was to sample out idiopathic patients for further genetic testing using next-generation sequencing (NGS) to determine possible genetic background.

## Patients and methods

### Patients

Patients aged 18 years or more, referred to the Haematology outpatient clinic, University Medical Centre (UMC) Ljubljana, between March 2011 and April 2019 were enrolled. Clinical data and blood counts were collected from the medical records. Men with Hb > 185 g/L and/or Hct > 0.52 and women with Hb > 165 g/L and/or Hct > 0.48 twice in an interval of ≥ two months were included. The study was approved by the National Medical Ethics Committee, Ministry of Health of the Republic of Slovenia; approval no. 115/07/15 (0120–198/2015–4, 0120–287/2019–4).

### Study design

**Fig. 1:**
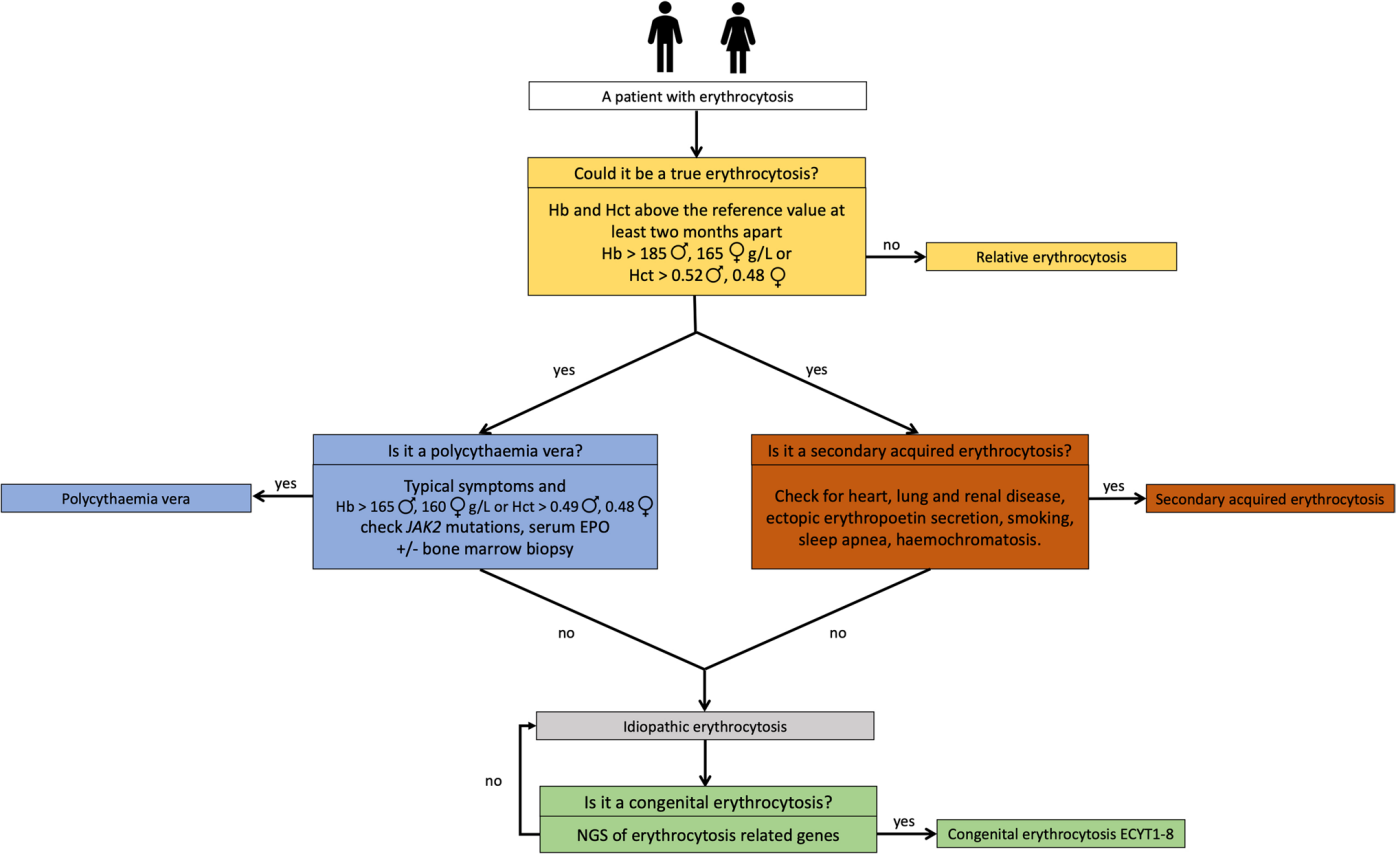
Simple three-step clinical algorithm for the diagnosis of patients with erythrocytosis

To characterize the type of erythrocytosis in our study population, we used a three-step diagnostic algorithm. The simplified schematic version is presented in Fig. 1. In the first step, only patients with two CBC measurements more than two months apart were included, thus assuming that the erythrocytosis is true (not relative).

In the second step, we confirmed or excluded PV patients by checking *JAK2* mutations (V618F and exon 12 when indicated) and serum EPO level. In patients with a subnormal EPO level and absent *JAK2* mutations, bone marrow histology was performed. The diagnosis of PV was evaluated according to the 2016 WHO diagnostic criteria [17]. Variant *JAK2* c.1849G > T (p.V617F) was detected by allele-specific PCR using DNA isolated from peripheral blood granulocytes as previously reported [18]. *JAK2* exon 12 variants were determined by Sanger sequencing after the mutation was detected by HRM analysis [19]. Serum EPO levels were determined by Solid Phase Sandwich ELISA test (R&D Systems, Inc., Abingdon, UK). At the same time, SE was confirmed or excluded by investigating the patient history, clinical, laboratory data, and the results of other diagnostic procedures. The following data were collected: patient's comorbidities (especially lung, heart, renal diseases), obesity, smoking, pulse oximetry, laboratory parameters (biochemistry, liver and kidney function, iron status, blood gases analysis with measurement of carboxyhaemoglobin (CO-Hb) and methaemoglobin (Met-Hb), oxygen pressure at 50% Hb saturation (p50), imagining techniques, polysomnography in snoring or obese patients with suspicion of obstructive sleep apnoea (OSA). In patients who demonstrated increased iron stores the most frequent mutations in the homeostatic iron regulatory genes (*HFE)*, namely c.845G > A (p.C282Y), c.193A > T (p.S65C), and c.187C > G (p.H63D), were determined by allele discrimination assays [20]. Haemoglobin electrophoresis was performed if congenital erythrocytosis was suspected. Treatment data with phlebotomies and antiplatelet agents and thrombotic complications were also collected from patients’ medical records.

In the third step, we identified IE patients. They were further referred for genetic testing using targeted next-generation sequencing (NGS). Selected patients who signed informed consent for participation underwent a targeted gene panel analysis that covered 24 erythrocytosis-associated genes [21, 22].

### Statistical analysis

In the statistical analysis, the median and interquartile range were used to describe the central tendency and variability of continuous variables. Frequencies were used to describe the distribution of categorical variables. A nonparametric Mann–Whitney test was used to compare continuous variables, while a Fisher Exact test was used to compare categorical variables. A *p*-value of < 0.05 was considered statistically significant. Statistical analysis was performed using R Statistical Software (version 3.5.1; R Foundation for Statistical Computing, Vienna, Austria) [23].

## Results

### Patients

A total of 126,284 CBCs were performed in adult patients at Haematology outpatient clinic, UMC Ljubljana, between March 2011 and April 2019. Among these, 655 patients met the criteria for true erythrocytosis. The criteria for non-clonal erythrocytosis were fulfilled by 116 patients; 75 (65%) men and 41 (35%) women. Median age was 51.4 years (41.1–62.9) and 60.7 (54.4–68.2), in men and women, respectively. Women were significantly older than men (*p* = 0.002). Median Hb and Hct at diagnosis were 184 g/L (181–192), 173 g/L (170–180) and 0.53 (0.53–0.55), 0.51 (0.50–0.53) for men and women, respectively. There were no differences in EPO levels between men and women (9.0 (6.1–11.2) vs 9.9 (7.3–17.0) U/L).

### Patient groups

After a complete review of the patients’ medical records in 34/116 (29%) patients a SE was determined, in 15/116 (13%) erythrocytosis remained idiopathic (IE) despite all relevant investigations performed, but 67/116 (58%) patients stayed incompletely characterized (ICE) as their condition did not seem clinically relevant for the treating haematologists to refer them to additional investigations. The investigation frequency performed and the rate of pathological results are presented in Table 1.

**Table 1:** Investigations performed/habits inquired in patients with non-clonal erythrocytosis (N = 116)

Investigations performed/habits inquired	Patients with investigations performed/habits inquired (number/% patients)	Abnormal results	
		*Decreased*	*Increased*
*JAK2* exon 14 V617F†	116 (100%)†	0 (0%)	
*JAK2* exon 12†	71 (61%)†	0 (0%)	
Ferritin	112 (97%)	3 (3%)	32 (28%)
Erythropoietin	111 (96%)	2 (2%)	16 (14%)
Transferrin saturation	110 (95%)	5 (5%)	17 (15%)
Pulse oximetry	73 (63%)	14 (19%)	-
Smoking	92 (79%)	45 (49%)*	
COHb measurement	24/45 active smokers (53%)	-	6 (25%)
Blood gas analysis	55 (47%)	-	
Snoring	39 (34%)	-	
Polysomnography	17/31 patients who reported snoring (55%)	13 (76%) **	
Hb electrophoresis	14 (12%)	0 (0%)	
p50	6 (5%)	0 (0%)	0 (0%)
*HFE*	24/38 patients with increased ferritin ortransferring saturation (63%)	1(4%)	

^†^Absent *JAK2* V617F mutation was an inclusion criteria. *JAK2* exon 12 analysis was not mandatory, however, if determined, it had to be negative

^*^Active smoking; **Confirmed sleep apnea

Exon 12 testing frequency was quite high with 61% of our patient population. More than 90% of patients had EPO, ferritin and transferrin saturation tested. Pulse oximetry was performed in only 63% and blood gas analysis in 47% of patients. Not all patients (79%) were asked about smoking and in only 53% of active smokers COHb was performed. Measurement of p50 was performed in only 6 (5%) patients. 34% of patients were asked about snoring. In half of them polysomnography was performed and OSA confirmed in 76%. Hb electrophoresis was performed in 12% of patients.

In the majority of patients, EPO was within the normal range (3.3–16.6 IU/L), it was increased in only 14% of patients. EPO level was higher in SE group but not significantly.

Table 2 compares laboratory characteristics of our patient groups: SE, IE and ICE; patients with IE were significantly younger than patients with SE and had significantly lower Hb and Hct values than patients with SE. The tobacco abuse and different comorbidities, associated with SE in our patient population are shown in Table 3. Most frequently SE was attributed to obstructive sleep apnoa, smoking and lung diseases.

**Table 2:** Characteristics and differences among patients with secondary erythrocytosis (SE), idiopathic erythrocytosis (IE) and incompletely characterized erythrocytosis (ICE) (N = 116)

Laboratory characteristics	Median (25–75%)			
	*Patients with SE (N* = *34)*	*Patients with IE (N* = *15)*	***p* value** (SE vs IE)	*Patients with ICE (N* = *67)*
Age (years)	57.7 (47.3–63.8)	41.1 (35.4–52.0)	**0.01**	58.0 (45.6–67.9)
Male (N)/female (N)	25/9	12/3	0.73	38/29
Haemoglobin (g/L)	189 (182–197)	182 (176–183)	**0.02**	180 (172–185)
Haematocrit	0.55 (0.53–0.58)	0.52 (0.52–0.53)	**0.002**	0.53 (0.51–0.54)
Erythropoietin (IU/L)	9.8 (7.6–17.2)	8.2 (6.1–12.5)	0.22	8.6 (6.2–11.3)

Haemoglobin and haematocrit in the table reflect the patient's first measurement before phlebotomies. Normal range of erythropoietin is 3.3–16.6 IU/L

**Table 3:** Diseases and habits causing secondary erythrocytosis in a group of patients with secondary erythrocytosis (N = 34)

Disease or habit	Number and frequencies of patients with SE
Lung disease (COPD) with saturation < 92%	6 (18%)
Cardiovascular disease (right-to-left cardiovascular shunts)	2 (6%)
Neurological disorders affecting respiratory function	2 (6%)
Obstructive sleep apnoea	13 (38%)
Active smoking with COHb ≥ 5%	7 (21%)
Kidney disease (RCC, renal cysts, polycystic kidney disease)	6 (12%)
Tumor with ectopic EPO secretion	2 (6%)*
Hemochromatosis confirmed with Sanger sequencing	1 (3%)
Drugs	2 (6%)

Some patients had more than one possible cause

^*^Hemangioblastoma, unspecified frontal tumour

*SE*, secondary acquired erythrocytosis; *COHb*, carboxyhaemoglobin; *COPD*, chronic obstructive pulmonary disease; *EPO*, erythropoietin; *RCC*, renal cell carcinoma

### Treatment and thrombotic complications

The treatment choice in selected patient groups is presented in Table 4. About 70% of patients in all three groups received aspirin. Phlebotomies were performed at least once in 56, 53 and 40% of patients in the SE, IE and ICE group, respectively. Among 15 patients with IE, 3 (20%) had thrombosis, whereas, in a group of 34 patients with SE, there were 4 patients (12%) with thrombosis; between the two groups no significant differences were found. In ICE group 10/67 patients (15%) had a thrombotic event.

**Table 4:** Treatment choice in patients with non-clonal erythrocytosis (N = 116)

	All patients (N = 116)	Patients with SE (N = 34)	Patients with IE (N = 15)	Patients with incompletely characterized erythrocytosis (N = 67)
Phlebotomies after exclusion of PV	54 (47%)	19 (56%)	8 (53%)	27 (40%)
Prescribed aspirin	82 (71%)	25 (74%)	10 (67%)	47 (70%)

*PV*, polycythaemia vera; *SE*, secondary acquired erythrocytosis; *IE*, idiopathic erythrocytosis

### NGS analysis results

Among 15 patients with IE, 13 underwent NGS analysis for congenital erythrocytosis. A pathogenic variant c.1609G > A (p.Gly537Arg) in the *EPAS1* gene, indicative of congenital erythrocytosis type 4 (ECYT4) was identified in one patient with elevated EPO levels. No other known disease-causing variants for congenital erythrocytosis were found.

## Discussion

Erythrocytosis frequently represents a challenge for the physician. While PV management has been well established [24], non-clonal erythrocytosis attracts less interest in the clinical and scientific community [12, 25]. Therefore, many *JAK2* negative erythrocytosis patients with erythrocytosis and with no apparent secondary cause for erythrocytosis do not have an adequate follow-up. Some haematologists even stop erythrocytosis work-up when PV is ruled out.

The three-step diagnostic algorithm, we used to retrospectively check diagnostic procedures performed in our patients, is a simple tool developed recently at our department to guide haematologists to proper erythrocytosis classification. In the first step, it reminds us which level of Hb and Hct warrants further investigation as well as that erythrocytosis can be relative. As RCM measurement as a gold standard for absolute erythrocytosis confirmation is not available in our centre and usually not elsewhere [26], we used two separate CBC measurements at least two months apart to confirm true erythrocytosis. Nevertheless, in the study of Johansson et al., where patients had RCM carried out, only 35% of men with Hb above 185 g/L and 65% of women with Hb above 165 g/L had absolute erythrocytosis [2, 27]. On the other hand, some patients with absolute erythrocytosis could be missed in our study due to inclusion requirement of two CBC.

In the second step of the algorithm, clonal erythrocytosis was ruled out and secondary causes were reviewed. *JAK2* genetic testing was performed in all of our patients and serum EPO level in 96%. After a thorough documentation review of all patients secondary erythrocytosis was found in 34/116 (29%) patients, 15/116 (13%) remained idiopathic despite adequate diagnostics, whereas a quite high number of patients, 67/116 (58%) would need further investigations for characterisation of erythrocytosis according to modern diagnostic algorithms [2–4, 11, 28]. It seems that patients with non-clonal erythrocytosis were not considered at high risk for complications and therefore were not referred for further investigations. It also seems that haematologists are less familiar or less interested in diagnostic algorithms of non-PV erythrocytosis and/or that non-clonal erythrocytosis is often trivialized [12, 25].

Fourteen percent of our patients had increased serum EPO which distinguishes secondary from primary erythrocytosis [5, 8]. The majority of patients had EPO within the normal range between 3.3 and 16.6 IU/L. This is surprising, as normal EPO level is inappropriate for a raised Hb and Hct [2]. As expected, patients with IE had lower EPO compared to patients with SE, but differences were not significant, probably due to low numbers. Additionally, a large number of ICE patients precludes us from interpreting EPO levels. In two patients, brain tumours were found (hemangioblastoma and unspecified frontal tumour). Furthermore, in three patients renal cysts were identified. Tumours with inappropriate EPO production and secondary erythrocytosis described in the literature are cerebellar hemangioblastomas, meningiomas, pheochromocytoma, uterine leiomyomas, parathyroid adenomas, hepatocellular carcinoma, and renal cell carcinoma [4, 29]. Renal cysts are also a known cause of EPO elevation [30, 31]. Two patients with renal cell carcinoma and erythrocytosis had normal EPO levels, a surprising but not an isolated finding [32]. A patient diagnosed with ECYT4 also had elevated EPO, although that is not necessarily the case in ECYT 4 [3, 33], which can present with normal, elevated, or decreased EPO [3].

Iron deficiency may sometimes mask erythrocytosis and mislead the diagnostic procedures. Both parameters of iron stores, ferritin and transferrin saturation should be measured in hypochromia with microcytosis or reactive thrombocytosis in CBC [10, 34]. Furthermore, in iron overload patients with increased ferritin (women ≥ 150 to 200 ng/mL and men ≥ 200 to 300 ng/mL) and transferrin saturation ≥ 45%, genetic testing for hereditary haemochromatosis is warranted as erythrocytosis sometimes, yet rarely, appears in haemochromatosis patients [35, 36]. Among 24 patients tested for common *HFE* mutations using Sanger sequencing, only one patient was diagnosed with hereditary haemochromatosis type 1 (HFE1), thus classified as SE patient. Recently, it was reported that patients with IE had a higher incidence of heterozygous *HFE* mutations, but possible relationship between hereditary haemochromatosis and erythrocytosis necessitates further evaluation [37, 38].

In 20% of our patients, smoking history was not taken, almost 40% have not had standard pulse oximetry performed and only a third were asked about snoring. As a result, fewer blood gas analyses and polysomnography testing were performed. Polysomnography is an investigation of choice when OSA is suspected. It should be performed in advanced cardiopulmonary disease, in patients with a higher likelihood of central apnoea, including those with heavy snoring, daytime somnolence, and/or increased BMI above 30 kg/m^2^ [24]. As smoking and OSA are the two most common causes of secondary erythrocytosis, also in our study group, patients would need such verification [4, 28]. P50 determination and Hb electrophoresis were done in 5 and 12% of our patients, respectively, with no pathological results found. If congenital erythrocytosis is suspected, p50 could be measured since it is a quick, simple, and inexpensive test done by most blood gas analysers [39]. However, the sensitivity and specificity of p50 in venous blood gases are low [40], and repetition for confirmation is indicated. With the advent of NGS and much broader diagnostic range available, p50 could be omitted as also suggested by others [41].

In the third step of our diagnostic algorithm, after excluding all known causes, 15 IE patients were identified. In such patients, especially those with a positive family history or erythrocytosis since childhood, Sanger sequencing of 9 genes involved in erythrocytosis can be used to assess a possible genetic background of erythrocytosis. It is known that by sequencing these nine genes only in around 30% of IE patients known pathogenic variants in causative genes for ECYT 1–8 are identified [3, 21]. NGS analysis has recently been shown to be a promising tool in finding new disease-driven variants [21]. At our centre, neither Sanger nor NGS had been available for congenital erythrocytosis until 2019. Among 13 IE patients from our study who underwent NGS analysis, one was diagnosed with congenital erythrocytosis type 4 (ECYT 4), which is indeed a very rare disease [41], described so far in approximately 50 patients worldwide [42].

The treatment guidelines for non-clonal erythrocytosis are not clear [24, 26]. This is in contrast with PV, where maintenance of Hct below 0.45 with phlebotomies, antiplatelet drugs, and cytoreduction in high-risk patients are the mainstays of treatment. As demonstrated in Table 4, phlebotomies were performed in 56%, 53% and 40% of our patients in SE, IE and ICE group, respectively. Approximately 70% of patients in all three groups were prescribed aspirin. Thrombotic events were documented in 12, 20 and 15% of SE, IE and ICE patients, respectively. British Society for Haematology guidelines for SE suggests that phlebotomy should be considered in patients with a recent thrombotic event, additional risk factors for thrombosis, or Hct higher than 0.56 [41, 43]. As erythrocytosis is a compensatory mechanism in these patients, the benefits and potential harms of phlebotomies should be considered [2]. Treatment decisions in our study group were made on a case-by-case basis, after an individualized risk–benefit assessment and depending on the physician’s belief. Rumi et al. reached a similar conclusion [26]. The main focus in non-clonal erythrocytosis, especially SE, should be identification of the cause and specific management, including discontinuation of a poor lifestyle habit such as smoking [43]. Randi et al. demonstrated that in a larger cohort of 78 patients with IE, 37 (47%) and 43 (55%) of the patients were treated with phlebotomies and aspirin, respectively [25]. According to British guidelines, patients with IE should be prescribed aspirin according to their cardiovascular risk factors, while phlebotomies can be used only in selected cases, but no target Hct is proven optimal [41]. Patients with congenital erythrocytosis differ in their need for phlebotomies. For instance, in patients with increased oxygen affinity hemoglobinopathies (ECYT 6, 7, 8), erythrocytosis is a compensatory mechanism, and phlebotomies could be harmful [8]. All in all, the type of congenital erythrocytosis, history of thromboembolic events, patients’ symptoms, and their alleviation after the procedure should be taken into consideration when deciding for phlebotomies [41].

In conclusion, our retrospective study gives us insight into a real life management of patients with non-clonal erythrocytosis. A large proportion of our patients remained inadequately characterized and consequently very likely insufficiently managed. We showed that thrombosis is not a rare event even in patients with non-clonal erythrocytosis. Also, diagnosing a neoplasm causing non-clonal erythrocytosis should always be considered. The three-step algorithm is a useful tool for everyday clinical evaluation of erythrocytosis. Secondary causes of erythrocytosis should be systematically checked and IE patients referred for genetic testing. Only patients with known aetiology of erythrocytosis can be properly managed.

## Data Availability

The data that support the findings of this study are available from the corresponding author upon reasonable request.
